# Factors that influence Puerto Rican's intention to get the COVID-19 vaccine

**DOI:** 10.1016/j.rcsop.2022.100106

**Published:** 2022-01-22

**Authors:** Page D. Dobbs, Emily Herrmann, Charlie Vidal, Daniela Ameijeiras Mena, Ches Jones

**Affiliations:** a Department of Health, Human Performance and Recreation, University of Arkansas, Fayetteville, AR, USA; b Puerto Rico Public Health Association, Utuado, Puerto Rico

**Keywords:** COVID-19, Vaccine hesitancy, Puerto Rico, Decision making

## Abstract

Despite Latino Americans having been found to be disproportionally affected by COVID-19, they report higher hesitancy to receive the COVID-19 vaccine than non-Hispanic whites. The purpose of this study was to examine factors that influence Puerto Rican's intentions to get the COVID-19 Vaccine. A sample (*n* = 173) of people who currently lived in Puerto Rico were recruited to complete an online, cross-sectional survey about their intention to receive the COVID-19 vaccine. Demographics and vaccine hesitancy were assessed, and logistic regressions explored relationships between variables and intention to get the COVID-19 vaccine when it became available. When controlling for covariates, 30–49-year-olds (aOR = 0.12) and those who had refused a vaccine in the past (aOR = 0.07) had lower odds of vaccine uptake than those between 18 and 29 years and who had not previously refused a vaccine, respectively. Those who had completed at least a 4-year college degree (aOR = 6.78) had greater odds of intending to get vaccinated than their counterparts. Health education campaigns about COVID-19 in Puerto Rico should be tailored to working-age adults who may mistrust information they have heard about the vaccine. Messages could be tailored to preferred communication channels to decrease vaccination hesitancy.

## Highlights

• Puerto Ricans between 18 and 29 years were more likely to get vaccinated than 30–49-year-olds.
• Puerto Ricans who had previously refused a vaccine had lower odds of getting the Covid-19 vaccine.
• Puerto Ricans with a 4-year college degree+ were more likely to get vaccinated.

## 1 Introduction

The first cases of the coronavirus disease 2019 (COVID-19) were discovered in China in 2019. Within months, COVID-19 became the largest pandemic in the last 100 years, infecting more than 248 million people and killing more than five million people worldwide, as of November 2021.^1^ Side effects of the virus vary from a fever to severe complications, including acute respiratory distress syndrome, which can result in hospitalization and even death in severe cases.^2^ Research suggests that among hospitalized patients, those living with underlying chronic illnesses (e.g., diabetes, obesity), who are older, and who are male are at greater risk of experiencing severe side effects from COVID-19 and being hospitalized than their counterparts.^3^

The U.S. has been the most highly affected country, with more than 45 million COVID cases and over 740,000 deaths, accounting for more cases and deaths than any other country in the world.^4^ Puerto Rico is an unincorporated territory of the U.S. and is home to primarily a Latino population, who have been found to be disproportionally affected by COVID-19, with higher morbidity and mortality rates than their White counterparts.^5^^,^^6^ The first cases of COVID-19 were first reported in Puerto Rico between March 9–13, 2020, and as of November 3, 2021, the Puerto Rico Department of Health reported there to have been 152,049 confirmed cases and 3239 deaths attributed to COVID-19.^7^ Although Latino Americans have lower mortality rates attributed to other respiratory illnesses (i.e., influenza and pneumonia) than non-Hispanic Whites,^8^ this group may be particularly vulnerable to COVID-19 due to social, cultural, economic, and environmental factors that increase their risk for many chronic diseases.^9^

By March 2021, the U.S. Food and Drug Administration (FDA) approved the distribution of three COVID-19 vaccines. With elevated COVID-19 cases in the U.S., compared to other countries,^1^ there remains the need to vaccinate adults and children,^10^ particularly among vulnerable populations.^5^ Despite the undue burden COVID-19 has had on minority populations, initial research suggests U.S. Latino adults have lower vaccination rates than non-Hispanic Whites^11^ and may be hesitant to receive the vaccine,^12^ with a recent study suggesting that Latino Americans are waiting to see how the vaccine affected others before getting it themselves.^13^ This differs from those living in Latin American (e.g., Brazil, Ecuador, and Mexico) where vaccine acceptance rates have been reported to be as high as 70%.^14^^,^^15^ Thus, vaccine hesitancy is an increasingly important public health concern for U.S. Latino populations, particularly for COVID-19 as the Delta Variant has increased hospital rates and deaths worldwide with most cases found among those who were not vaccinated.^16^

Theoretical models, such as the Health Belief Model,^17^ have been used in recent research to help explain attitudes toward the vaccine and vaccine hesitancy for the COVID-19 vaccine during the pandemic.^18^, ^19^, ^20^, ^21^ Perceived barriers and benefits have been found to be associated with vaccine hesitancy and vaccination uptake intention,^18^ with barriers decreasing one's intention to receive the COVID 19 vaccine and benefits increasing their vaccination uptake intention.^20^ With approximately 57% of the U.S. indicating intention to receive the vaccine and greater hesitancy among Latino Americans than non-Hispanic whites,^22^^,^^23^ it is important to understand what may prevent this population from getting vaccinated. Thus, fear-based theoretical models and theories can help to understand what factors may impact behavioral intention and behavior.

While research has focused on preventative practices^24^^,^^25^ and morbidity/mortality^26^^,^^27^ among predominately Latino populations (e.g., Puerto Ricans), there remains a gap in research regarding vaccination rates among Puerto Ricans and factors that might influence this vulnerable population's intention to receive the COVID-19 vaccine. Therefore, it was the purpose of this study to explore factors associated with Puerto Ricans' intentions to get the COVID-19 Vaccine.

## 2 Methods

### 2.1 Participants and procedures

Those who currently lived in Puerto Rico and were 18 years of age and older were invited to participate in an anonymous, online, cross-sectional survey between January 26 and March 17, 2021, around the time that vaccines were first available. Participants were recruited via social networks (e.g., Facebook, Twitter, Reddit). No incentives were used to recruit participants. Informed consent was obtained before participants completed the survey, which was offered in both English and Spanish via Qualtrics. All study procedures were approved by the [Blinded for Review] Institutional Review Board.

### 2.2 Measures

Demographic items measured participants' age, sex, marital status, educational attainment, race/ethnicity and asked if the participant had any current chronic diseases or was a healthcare worker. These demographic items were identified from previous COVID-19 related research.^28^^,^^29^ Participants were also asked about the perceived risk of contracting COVID-19, vaccine hesitancy, and intention to get the COVID-19 vaccine. Marital status was dichotomized into “married” or “single” with those who were single, widowed, or divorce categorized as “single”. Race/ethnicity was dichotomized into “Hispanic/Latino” and “Other”; those who identified as White, Black or African American, Native American or American Indian, Asian/Pacific Islander, and Other were categorized as “Other” due to frequencies.

Vaccine hesitancy was assessed using two items used in previous research: (1) “Have you ever refused a vaccine for yourself or a child because you considered it as useless or dangerous?” and (2) “Have you ever postponed a vaccine recommended by a physician because of doubts about it?”^28^ Participants provided yes/no dichotomous responses to each item. Intention to receive the vaccine was measured by asking participants' “Do you plan to receive the coronavirus vaccine when it is available to you?” Responses were measured on a 5-point Likert scale. To dichotomize responses for non-parametric testing, we grouped “Yes definitely” and “Yes possibly” into one group (yes) and “I don't know”, “No possibly”, and “Definitely not” were categorized into another group (no). I don't know was dichotomized with ‘no’ responses due to overall response rates (see Table 1). Those who indicated they did not plan to receive the vaccine were asked to provide an open-ended response with at least one reason for this decision.

**Table 1:** Demographic characteristics: Puerto Rican sample about COVID 19 vaccine (*N* = 173).

		N	%
Sex			
	Woman	119	68.8
	Man	54	31.2
Age			
	Under 30 years	59	34.1
	30–49 years	60	34.7
	50–64	38	22.0
	65+ years	16	9.2
Marital Status			
	Single, Widowed, Divorced	113	65.3
	Married	60	34.7
Educational Attainment			
	Less than 4-year college degree	38	22.0
	4-year degree or higher	135	78.0
Race/Ethnicity			
	Hispanic or Latino	142	82.1
	Other (white, black, other)	31	17.9
Chronic Disease			
	No	120	69.4
	Yes	53	30.6
Are you a healthcare worker?			
	No	134	77.5
	Yes	39	22.5
Household Member 65 years or Older			
	No	76	43.9
	Yes	97	56.1
Are you afraid of COVID 19?			
	No	48	27.7
	Yes	125	72.3
Do you feel at risk for contracting COVID 19?			
	No	53	30.6
	Yes	120	69.4
Have you ever put off getting a vaccine recommended by your doctor?			
	No	112	64.7
	Yes	61	35.3
Have you ever refused to get a vaccine or give it to your children because you thought it was useless or dangerous?			
	No	149	86.1
	Yes	24	13.9
Do you plan to receive the coronavirus vaccine when it is available to you?			
	Yes definitely	128	74.0
	Yes possibly	20	11.5
	I don't know	10	5.8
	No possibly	4	2.3
	Definitely not	11	6.4

### 2.3 Data analysis

Of the 285 participants who were recruited to complete the survey, 17 did not meet eligibility criteria, 63 did not provide consent to participate or did not continue to complete the survey after reading the consent page, and 30 did not complete relevant variables for the current analyses. During the analysis, we identified two participants who misinterpreted the open-ended item that asked their reason for not intending to get vaccinated by answering that they had already received it. Subsequently, we removed these two responses, and the final sample included 173 participants. Descriptive statics were reported, and logistic regressions examined relationships between independent variables (i.e., demographics variables, perceived risk of contracting COVID-19, and vaccine hesitancy) and intention to get the COVID-19 vaccine (dependent variable). After removing one item (afraid of COVID-19) for multicollinearity issues with the item that measured the risk of contracting COVID-19, we determined there to be no multicollinearity issues (tolerance ranged from 0.71–0.93 and variance inflation factor [VIF] ranged from 1.05–1.4; numbers closer to 1 indicting stronger independence and less multicollinearity).^30^ Odds ratios (OR) and adjusted odds ratios (aOR) were reported along with 95% confidence intervals (95% CI). All confounding factors included in the multivariate logistic regression model are reported in Table 2, where the adjusted odds ratio adjusts for the other factors included in the regression model. Using SPSS 26 software,^31^ alphas for all analyses were set a priori at 0.05. Variability was reported using Nagelkerke R.^2^ Finally, open-ended responses were analyzed by grouping similar responses together into categories, a technique used in prior research that explored responses to an open-ended item.^32^^,^^33^

**Table 2:** Factors associated with Puerto Ricans intention to get the COVID-19 Vaccine (N = 173).

Variables		OR	95% C.I.	aOR	95% C.I.
Sex					
	Female	1.00		1.00	
	Male	2.68	0.87, 8.23	1.63	0.43, 6.23
Age					
	18–29 years	1.00		1.00	
	30–49 years	0.10	0.02, 0.44	0.12	0.02, 0.76
	50–65 Years	0.19	0.04, 0.98	0.33	0.04, 2.65
	65+ years	0.53	0.05, 6.20	0.54	0.03, 11.06
Marital Status					
	Single	1.00		1.00	
	Married	0.52	0.22, 1.23	0.75	0.23, 2.45
Educational Attainment					
	Less than 4-year college degree	1.00		1.00	
	4-year college degree or more	4.33	1.78, 10.56	6.78	1.96, 23.43
Race/Ethnicity					
	Other (non-Hispanic White, Black)	1.00		1.00	
	Hispanic or Latino	2.01	0.76, 5.33	1.80	0.47, 7.00
Chronic Disease					
	No	1.00		1.00	
	Yes	0.75	0.31, 1.83	0.65	0.17, 2.52
Healthcare Worker					
	No	1.00		1.00	
	Yes	2.36	0.67, 8.34	2.25	0.42, 12.24
Household Member 65 years or Older					
	No	1.00		1.00	
	Yes	3.21	1.30, 7.90	2.15	0.66, 7.08
Risk of COVID-19					
	No	1.00		1.00	
	Yes	1.08	0.43, 2.68	1.56	0.33, 4.11
Put off Vaccine					
	No	1.00		1.00	
	Yes	0.37	0.15, 0.87	0.50	0.15, 1.63
Refused Vaccine Self/Children					
	No	1.00		1.00	
	Yes	0.10	0.04, 0.26	0.07	0.02, 0.27
R^2^				0.473	

## 3 Results

### 3.1 Demographics

The majority of our Puerto Rican participants identified as female (68.8%), Hispanic or Latin (82.1%), and were single (65.3%). Further, 59 (34.1%) participants were less than 30 years of age, and 53 (30.6%) reported having a chronic disease. Most were not healthcare workers (77.5%), had completed a 4-year college degree (78.0%), and lived with someone who was 65 years or older (56.1%). See Table 1 for frequencies.

### 3.2 COVID perceptions

When asked about COVID-19, 72.3% of the sample reported they were afraid of the disease, and 69.4% felt at risk for contracting it. Further, 35.3% of participants reported they had put off a vaccine recommended by their doctor in the past, and 13.9% of participants had refused a vaccine for themselves or their child because they thought it was useless or dangerous.

### 3.3 Associations between variables and intention to get COVID-19 vaccine

Variables associated with intention to get the COVID-19 vaccine included age, educational attainment, putting off getting a vaccine and refusing to get a vaccine. Those between 30 and 49 (OR = 0.10; 95% CI = 0.02–0.44) and 50–65 (OR = 0.19; 95% CI = 0.04–0.98) years had lower odds of intending to receive the COVID-19 vaccine than 18–29-year-olds (see Table 2). Those who have previously postponed a vaccine (OR = 0.37; 95% CI = 0.15–0.87) or had ever refused a vaccine (OR = 0.10; 95% CI = 0.04–0.26) had lower odds of intending to receive the COVID-19 vaccine than their counterparts. Those who had completed a 4-year college degree or higher were over four times as likely (OR = 4.33; 95% CI = 1.78–10.56) to intend to get vaccinated as those who completed less than a 4-year college degree. Further, those who lived with someone who was 65 years of age or older (OR = 3.21; 95% CI = 1.30–7.90) had higher odds of intending to get the vaccine than their counterparts.

After adjusting for all other variables, age, educational attainment, and vaccine refusal for oneself or child were found to be independently associated to intention to get the COVID-19 vaccine. Those between 30 and 49 years (aOR = 0.12; 95% CI = 0.02–0.76) had lower odds of intending to get the vaccine when compared to 18–29-year-olds. Those who have ever refused a vaccine (aOR = 0.07; 95% CI = 0.02–0.27) had lower odds of intending to receive the vaccine than their counterparts. Alternatively, those who had completed a 4-year college degree were more than six times as likely (aOR = 6.78; 95% CI = 1.96–23.43) as those who had not completed a 4-year college degree to intend to receive the vaccine. The model explained 47.3% of the variability of intention to get the COVID-19 vaccine.

### 3.4 Reasons for not intending to get COVID-19 vaccine

Three categories emerged from the open-ended responses about reasons to get the vaccine: trust, medical reasons, and other reasons. Among the 27 responses, the most common reason was trust issues (*n* = 16, 59.3%). Participants included responses including: “*I don't trust the vaccine as it is very premature*”, “My *body should not be a part of an experiment”, and “Possible second effects.”* Six (22.2%) participants reported having a medical condition that limited their ability to get the vaccine: *“I am allergic to various medicines.”* Lastly, five participants included various non-related reasons.

## 4 Discussion

We sought to explore factors that influenced Puerto Ricans' intentions to get the COVID-19 Vaccine. While studies are emerging about vaccine hesitancy and particular populations' intentions to get the vaccine, studies specific to Puerto Rico are limited to early interventions used to slow the spread of the virus,^25^ prevention practices,^24^ and morbidity, and mortality risks.^26^^,^^34^ Thus, understanding factors that may influence COVID-19 vaccine uptake intentions among this predominately Latino population is increasingly important, especially with U.S. Latino adults reporting greater vaccine hesitancy than non-Hispanic whites.^12^ Interestingly, we found the majority (85.5%) of our sample to report intention to receive the COVID-19 vaccine during a time when it was initially becoming available, findings that mirrored vaccine acceptance rates in Latin countries more than Latino populations living in the U.S.^14^^,^^15^

While vaccines have been recognized as one of the greatest public health accomplishments of the past 100 years,^35^ the spread of false safety information about vaccines via the Internet has created devastating public health challenges even before the COVID-19 vaccine was made available to the public.^36^ Some researchers attribute declines in vaccination rates to an Australian documentary about vaccines and autism, which has been studied via online debates following the release of the documentary^37^; however, others suggest that vaccine hesitancy may vary by social networks, where diffusion of information may exacerbate anti-vaccine sentiment within sub-groups of populations.^38^ Given the physical isolated state of Puerto Rico, such false claims about the COVID-19 could circulate among interpersonal networks quickly, which could increase health disparities among different sub-groups of Puerto Ricans (e.g., those with lower levels of educational attainment, working-aged adults). Alternatively, effective health communication may also be accepted more quickly among this population than their Latino American counterparts living in the U.S.

Consistent with previous studies among other populations,^39^ we found that greater educational attainment was associated with increased intention to get the COVID-19 vaccine. Further, similar to a study among working-age adults (defined as those 18–64),^40^ we found 30–49 year-olds were less likely than those less than 30 years to intend to receive the vaccine. Working-age adults may be less likely than younger and older populations to intend to be vaccinated against COVID-19. However, among our sample, those living with someone who was 65 years of age and older were associated with increased odds of intending to get vaccinated; however, it was not significantly related to vaccine uptake intention when adjusting for all other factors. Thus, those working to increase vaccinations rates in Puerto Rico may consider emphasizing family-based messages to working adults.

Interestingly, when we asked those who intended to refuse the vaccine for reasons that would prevent them from getting vaccinated, the most common responses were due to trust. Research suggests that U.S. Latino populations (and Black populations) report historical mistreatment in research (e.g., Tuskegee study) and feel as though their voice is ignored by medical professions, leading to mistrust in those developing and distributing the vaccine.^41^ However, other research suggests health professionals, academic institutions, and government agencies were the most trusted sources of information about COVID-19 and were trusted more than news media, family/friends, and social media.^42^ Although trust in sources sending information about the vaccine is important because of the influence it can have on intention to get vaccinated against COVID-19,^43^ it is unknown how U.S. Latino populations perceive information sources reporting about COVID-19.

Those living in Latin America who were identified as optimists were found to have lower levels of fear about COVID-19 while pessimists had higher levels of fear, which was found to be related to all information sources.^44^ While this helps to explain that excessive information about COVID may instill fear,^44^ this has not been explored among Latino populations living in American states and territories. Latino Americans who have been hospitalized report receiving misinformation about COVID-19, which may have put them at an increased disadvantage for not getting the vaccine and contracting COVID-19.^45^ Alternatively, as of September 2021, vaccination rates among Hispanic Americans had increased to rates similar to those of their non-Hispanic white counterparts.^46^ These findings may suggest that Latino Americans are gaining trust in the vaccine.

### 4.1 Limitations

Due to convenience sampling, no generalizations can be made about those living in Puerto Rico. Of our sample, 78.3% reported having a 4-year degree or higher, which is substantially higher than the 27.2% of Puerto Ricans reported to have a bachelor's degree or higher,^47^ further emphasizing the lack of generalizability of the data. Further, because all participants had social media, findings are not representative of consumers in the population without social media accounts. Participants were not recruited from local health centers, departments of health, and pharmacies due to COVID restrictions that prevented the research team from distributing materials in locations where participants would normally have waited; however, researchers should consider such methods as locations for advertising similar research when possible. Due to the cross-sectional nature of this study, findings do not imply causality. Some odds ratios had wide confidence intervals; thus, these results may be interpreted with caution.

## 5 Conclusions

Among our sample, age, educational attainment, and previous refusal of a vaccine were associated with intention to get the COVID-19 vaccine when it became available. Similar to other populations, working-age Puerto Ricans may be less likely to trust information about the vaccine, which could affect uptake. However, more research is needed to understand vaccine hesitancy among Latino populations as well as what type of information this population does trust. Further, higher educational attainment was associated with greater intentions to get the vaccine. Thus, those creating education material to increase vaccine uptake among those living in Puerto Rico should ensure health campaigns about the vaccine use appropriate literacy levels to increase trust among those with less education and are tailored to address concerns of disparate populations and those most hesitant to receive the vaccine.

## Funding details

This work was not funded.

## Disclosure statement

The authors report no conflicts of interest.
