# Erratum to “Factors that influence Puerto Rican's intention to get the COVID- 19 vaccine” [Exploratory Research in Clinical and Social Pharmacy 5C (2022) 100106]

**DOI:** 10.1016/j.rcsop.2022.100197

**Published:** 2022-10-22

**Authors:** Page D. Dobbs, Emily Herrmann, Charlie Vidal, Daniela Ameijeiras Mena, Ches Jones

**Affiliations:** a Department of Health, Human Performance and Recreation, University of Arkansas, Fayetteville, AR, USA; b Puerto Rico Public Health Association, Utuado, Puerto Rico

The publisher regrets that the section below was accidentally anonymized in the original published version of this article:

**“All study procedures were approved by the [Blinded for Review] Institutional Review Board”.**

This section should read:

“All study procedures were approved by the University of Arkansas Institutional Review Board.”

The publisher would like to apologise for any inconvenience caused.

